# Betting on the future: how to build antifragility in innovative start-up companies

**DOI:** 10.1007/s11846-023-00636-x

**Published:** 2023-05-12

**Authors:** Vincenzo Corvello, Alberto Michele Felicetti, Ciro Troise, Mario Tani

**Affiliations:** 1 Department of Engineering, University of Messina, Contrada di Dio, 98166 Messina, Italy; 2 Department of Mechanical Energy and Management Engineering, University of Calabria, Ponte P. Bucci 42C, 87036 Rende, Italy; 3 Department of Management, University of Turin, Turin, Italy; 4 Department of Economics, Management and Institutions, University Federico II, Naples, Italy

**Keywords:** Innovative startup companies, Antifragility, Resilience, Intellectual capital, Absorptive capacity, Innovation, M13, O34

## Abstract

While after a crisis, organizational robustness and resilience are associated with the return to pre-shock conditions, antifragility is characterized by the tendency to take advantage of the shock to improve the organization’s position. Understanding how to create antifragility is important to ensure rapid recovery from a crisis. This is especially true for innovative start-ups, which if they are particularly fragile also have the opportunity to improve their unstable situation by adapting to the new context. However, studies on the subject are rare. To fill this gap, a survey was carried out with 181 Italian innovative start-ups to investigate the relationship between antifragility and intangible resources, tangible surplus resources, and absorptive capacity. The results show that antifragility is supported by a combination of tangible and intangible resources that can help innovative start-ups thrive when other organizations succumb.

## Introduction

Crises such as the ongoing pandemic or the conflict in Ukraine have inevitably turned the spotlight on the importance of dealing with disastrous events (Kraus et al. 2013; Parameswar et al. 2021). The COVID-19 crisis raised awareness of market instability when exposed to systemic issues, highlighting the dramatic consequences of failing to be prepared (Henderson 2020; Kraus et al. 2020; Emami et al. 2021). These significant catastrophic events are sometimes called “black swans,” i.e. highly unlikely events that are very difficult to predict, and have disastrous impacts (Taleb 2005). However, it should be noted that these events occur more frequently than might have been expected.

Over the past 20 years, there have been several events recognized as black swans, e.g. the 9/11 disaster in 2001 (Taleb 2005), the global financial crisis of 2007–2009 (Siegel 2010), the UK’s BREXIT vote in 2016 (Blyth and Matthijs 2017), and, as mentioned, the COVID-19 pandemic and the war in Ukraine (Yarovaya et al. 2022). According to Taleb et al. (2009), black swan events are occurring more often since the world is increasingly becoming a complex system, with a dense network of relationships and interrelated factors.

Scholars and policymakers agree that these ongoing crises may be considered a test for other serious changes that could happen in the coming years (e.g. the exhaustion of natural resources, pollution, and climate change) (Chakraborty and Maity 2020). This highlights the greater need for preparedness of countries, society, and companies in order to face the upcoming challenges. Faced with these events, many businesses turn out to be fragile: they suffer severe damage or even disappear (Taleb 2012).

Recently, especially following the lockdown caused by COVID-19, great attention has been paid to the concept of resilience (Remko 2020; Chowdhury et al. 2021; Santoro et al. 2021) and antifragility (Nikookar et al. 2021). According to some scholars, the concepts of resilience and antifragility are closely related, and in some cases overlapping (Cavanagh 2017). However, there is an ontological difference makes two terms clearly distinct: antifragility implies the tendency of an organization to improve its own competitive position in the face of a crisis, while resilience does not (Munoz et al. 2022). While a resilient firm is able to absorb the shock and when the crisis is over can return to its prior state (Ivanov 2021), an antifragile firm benefits from disorder (Taleb 2012; Ramaezani and Camarinha-Matos 2020). Companies that embody this second capability are able to grow when exposed to disorders brought about by black swan events as they learn from such situations (Taleb 2012).

An organization is considered antifragile if its performance is said to be improved following a catastrophic event (Fang and Sansavini 2017). However, an improvement in company performance could be due to idiosyncratic and contingent situations (i.e. by accident). In fact, a company that occasionally responds positively to a crisis is not necessarily antifragile. Consistent with the majority of previous studies (Munoz et al. 2022), instead, we assume that antifragility is a capacity possessed by a company that enables it to positively respond to several types of disastrous phenomena, thus gaining a competitive advantage in the market (Taleb 2012).

Among the organizations that risk suffering the impact of a crisis, a category particularly worthy of attention is that of start-up companies. Various studies have addressed the role of start-ups as key players for growth (Rigtering and Behrens 2021; Sandulli et al. 2021; Kim and Lee 2022), acting as an essential driver for the economic sustainability of regional systems (Bresciani et al. 2021; Fernandes and Ferreira 2022). Start-ups, in other contexts are referred to as high-tech entrepreneurial ventures; in some cases overlapping with innovative small and medium-sized enterprises (SMEs) or young tech ventures (Kraus et al. 2019), they are fragile by their nature due to their small size (Gimenez-Fernandez et al. 2020).

Start-ups are carriers of great innovative potential, as well as the ability to create entire ecosystems around emerging technologies (Colombelli et al. 2016; Rudeloff et al. 2022). Start-ups are recognized as an important source of new jobs and a “flywheel” for national economies (Ammirato et al. 2020). For this reason, there has been a worldwide increase in government policies to support this kind of company. Governments have offered several relief packages and measures, including taxation support, economic support, loans, and special programs to support start-ups during crisis events such as the COVID-19 pandemic (Kuckertz et al. 2020).

The scientific literature on start-ups has mainly focused on aspects relating to their innovative performance (e.g. Steiber et al. 2020), placing less emphasis on other aspects, such as their ability to react to the crisis. Start-ups are often characterized by a high mortality rate, especially in the early stages of their lifecycle (Hyytinen et al. 2015). This mortality is even more pronounced in times of crisis (Stephens et al. 2021), thus resulting in the loss of a fundamental pillar for innovation systems.

On the other hand, they are flexible and innovation-based by definition and, thus, can adapt to rapidly changing contexts. They often find themselves competing with incumbents who hold advantageous positions, making it difficult for them to achieve significantly better results (Branicki et al. 2018). As long as the competitive environment remains stable, competition for start-ups remains difficult. A crisis, however, changes the conditions around the world. Antifragile start-ups find in this situation an opportunity to radically improve their situation.

Although much of the existing literature groups the possible positive reactions to shocks under the single concept of resilience, there are different modes of response, characterized by different enabling factors and dynamics (Ramezani and Camarinha-Matos 2020; Hilmann 2021). This implies the need for greater precision in the definition of these concepts, distinguishing one type of response from another and developing specific studies for each one (Simmie and Martin 2010; Martin 2012).

In this light, our study focuses on the enabling factors of antifragility, hypothesizing them on the basis of the analysis of the literature, as distinct from those of resilience. The need for research in this regard becomes even stronger in the case of start-ups, for which the concept of antifragility assumes a greater importance than that of resilience. These organizations, in fact, are structurally in a precarious situation (Gimenez-Fernandez et al. 2020); they can only react to a crisis by trying to exploit the new context to reach a new and better state of equilibrium. Existing research, focusing on incumbents, favors the concept of resilience and neglects factors of vital importance for start-ups.

Our research question can be formulated as follows: What factors support antifragility in innovative start-ups? We investigate this issue by means of a statistical survey of a sample of Italian start-up firms. It is important to understand the factors that promote antifragility in start-ups. Studies on antifragility, however, are rare. Although similar properties have been considered and investigated (e.g. evolutionary resilience), the specific phenomenon of firms aiming to improve their results during crises is less studied. In the context of start-ups, this is even more true.

For this reason, in this study we focus on antifragility and the factors able to promote this property in start-ups. We hypothesize that a relevant effect on antifragility is exerted by intellectual capital (Mubarik et al. 2021), the availability of slack resources (Leuridan and Demil 2021), and the absorptive capacity of the firm (Cohen and Levinthal 1990).

## Theoretical background

### From resilience to antifragility

Resilience and antifragility are two frequently used concepts to describe the important capabilities of firms to survive and thrive in unpredictable business environments. The concept of resilience has gained huge prominence in many disciplines over the last two decades (Koliou et al. 2020), ranging from psychological studies to other different contexts, such as ecology, risk management, disaster management, safety engineering, supply chain management, and business ecosystems (Bhamra et al. 2011; Ponomarov and Holcomb 2009). Resilience leverages the metaphor of a material’s ability to absorb energy and resume its initial form when the deforming force is removed (Carpenter et al. 2001). In economics and organizational studies, resilience in understood as the company’s ability to cope with unexpected events (Pettit et al. 2013). Studies on the reaction of companies to disasters describe a great variety of possible reactions and situations, ranging from passive resistance to response planning in order to adapt rapidly to the new situation (Martin 2012).

Of note, several definitions of resilience have been presented in the literature. Focusing on the adaptive capability of a system, some scholars have used the term “adaptive resilience” to describe a company’s ability to undergo a destabilizing shock, returning to a new state of equilibrium (not necessarily the same as the previous one), and adapting its configuration to the exogenous changes that have occurred in the surrounding environment (Chowdhury et al. 2019). Other authors have explored the concept of strategic resilience as the ability of a company to change and reinvent itself (Hamel and Valikangas 2003). This involves the need to reformulate long-term goals and the means to achieve them, due to substantial changes in the competitive environment (Shin and Park 2021).

Other works have focused on the operational aspects of systems, defining resilience as the ability to maintain operational continuity following destructive events, leveraging flexibility, the redundancy of resources, and buffer capacities (Essuman et al. 2020). These aspects of operational resilience are also known as robustness or disruption absorption, i.e. a firm’s ability to maintain the normal functioning of its operations in the face of disruptions (Chowdhury and Quaddus 2017).

Antifragility is an emerging concept that shares some common points with resilience. Similar to resilience, antifragility deals with the capacity to react to catastrophic events (Markey-Towler 2018) and the ability of an organization to readjust to new equilibrium configurations (similar to adaptive resilience) (Ruiz-Martin et al. 2018). Furthermore, as in the case of strategic resilience, it presupposes changes in the company’s products, markets, and long-term objectives (Blečić and Cecchini 2019). However, antifragility is not about robustness and the ability to passively resist sudden changes in the business environment (Abbas and Munoz 2021). Ultimately, the key difference lies in the implicit pursuit of obtaining an advantageous competitive position (Ramezani and Camarinha-Matos 2020; Munoz et al. 2022).

In brief, resilience represents the capability to absorb shocks and although temporarily changing to return to business as usual after such shocks (Bhamra et al. 2011). Antifragility, however, represents the capability of a system to absorb shocks and subsequently improve (Ramezani and Camarinha-Matos 2020). While a robust or resilient system resists failure and remains the same or recovers from failure, an antifragile system benefits from shocks by getting better (Lichtman et al. 2016; Munoz et al. 2022).

While studies on resilience in organizations are numerous, research on antifragility is still limited. In particular, studies on antifragility in small firms are rare (de Bruijn et al. 2020). The ability to respond to a crisis by transforming one’s business model and even improving is at the heart of antifragility (Blečić and Cecchini 2019; Conz and Magnani 2020). Antifragility is therefore a highly desirable property, but how to develop it is not yet well understood (Chroust et al. 2016).

In their literature review, Ramezani and Camarinha-Matos (2020) identified the skills that enable the creation of resilience and antifragility. These skills manifest in the three main phases of crisis management: *readiness*, i.e. the phase before the disaster; *response*, i.e. the actions implemented after the crisis; and *recovery*, referring to the activities that are adapted post-crisis.

Currently, organizations are increasingly exposed to unforeseen disastrous events, which are no longer an exception but have instead become increasingly frequent. The effects and consequences of these events are unpredictable, causing major damage to many companies by changing their planning capabilities and disaster response readiness (Gotham and Campanella 2010).

Focusing on start-ups, crises can become an opportunity if organizations have the ability to increase their competitiveness (Mendoza et al. 2018). However, to achieve this degree of success, they need to develop specific skills (Máñez et al. 2015). The characteristics of start-ups, such as flexibility and adaptability, are crucial to responding to a crisis (Branicki et al. 2018). Furthermore, start-ups are generally used to working in conditions of uncertainty linked to, for example, limited financial and human resources and making entrepreneurs more comfortable in conditions of uncertainty than large organizations. This can be an advantage as they are more likely to perceive the crisis as an opportunity (Branicki et al. 2018).

The main differences between the concepts of resilience and antifragility are summarized in Table 1.

**Table 1:** Conservative resilience, proactive resilience and antifragility

	Conservative resilience	Proactive resilience	Antifragility
Attitude towards change	Seek stability	Adapt to the new context	Adapt to the new context
Attitude towards performance	Maintain performance level	Maintain performance level	Improve performance level

### Start-ups in the face of crises: the role of intellectual capital

An innovative start-up is a small or medium-sized company that translates ideas and technologies into new products, services, processes, or business models (Brown et al. 2022). Start-ups are mainly involved in the process of discovering, developing, and implementing and/or maintaining a viable business model to exploit market opportunities (Ehrenhard et al. 2017).

Scholars and policy makers agree that start-ups play a crucial role both as an engine of economic growth, a driver of regional innovation, and a key contributor to social wealth (Eshima 2003; Honjo and Harada 2006). For these reasons, governments are increasingly having to pay attention to regulatory and/or governance aspects to support the growth potential of start-ups (Yang 2017).

Start-ups’ innovation activities differ significantly from those of traditional established firms, and their needs and methods of operation are different compared to mature companies (Criscuolo et al. 2012). In the reconstruction of a post-pandemic world and economy, their role as the vanguard of economic and technological development is expected to be central. Despite the current difficulties, start-ups have shown adaptability and, in many cases, have been able to rapidly generate new solutions to face the effects of the pandemic.

These companies, innovative by definition, have changed their business models to adapt them to the pandemic scenario (Kuckertz et al. 2020). However, given the risk and uncertainty associated with entrepreneurial endeavors and innovation-based activities, start-ups are more likely to be exposed during a period of crisis (Brown et al. 2022). It is necessary to know the internal and external factors that allow start-ups, especially in the early phases of their lifecycles, to overcome the high risk of mortality by removing barriers and threats linked to their small size (Gimenez-Fernandez et al. 2020).

Many studies have investigated the influence of internal resources on the survival of start-ups (Aspelund et al. 2005; Newbert et al. 2007; Garnsey and Leong 2008; Huang et al. 2012). Although most studies have drawn the conclusion that initial resources do indeed affect the survival and growth potential of new ventures (Aspelund et al. 2005), some have argued that it is not the mere possession but rather the exploitation of a firm’s resources that determines its performance (Newbert et al. 2007), and that entrepreneurial managers can build capabilities within a network of firms to alter their business environment (Garnsey and Leong 2008).

The basic hypothesis, therefore, is that the success of start-ups is determined by a combination of factors, both internal and external, and the organization is able to acquire during its development. Internal factors are the resources and capabilities of the start-up, encompassing the ability of the entrepreneurial team, financial resources, intangible assets (e.g. patents), and relationships and networking skills. Both internal and external intangible resources can be understood as intellectual capital, considering the well-known distinction between human, structural (or organizational), and relational (or social) capital (Edvinsson et al. 1997; Bontis 1998).

Human capital generally represents the resources created from the stocks and flow of knowledge shared among individual owners, managers, and employees within a firm (Pennings et al. 1998). The peculiarity of human capital is that it is not really “owned” by the company, as it is closely linked to the people who work there and can leave the company very easily (Campbell et al. 2012; Brymer et al. 2014). Scholars have widely acknowledged that human capital is a critical component of firm performance (Reed et al. 2006; Bendickson et al. 2017), particularly when human capital investments focus on knowledge and skills.

Relational (or social) capital is defined as “the sum of the actual and potential resources embedded within, available through and derived from the network of relationships possessed by an individual or social unit” (Nahapiet and Ghoshal 1998). Relational capital, as an intangible resource, is difficult for competitors to imitate and creates value for the firm through the communication and assimilation of individual-level knowledge, helping them achieve and sustain a competitive advantage (Barney 2001).

Structural (or organizational) capital represents the unique knowledge institutionalized and codified by a firm through policies and procedures, routines, processes, work systems, and management structures (Youndt and Snell 2004). Organizational capital is generally presented as intangible assets, practices, and processes related to acquiring and retaining talent, culture, leadership, the alignment of human resources with strategic goals, organizational design, along with the leadership’s role in transforming resources into a competitive advantage (Eisfeldt and Papanikolaou 2013).

Many works have emphasized the role of intellectual capital as a determinant of SMEs’ success (Hormiga et al. 2011; Vrontis et al. 2021). Scholars have found a positive relationship between intellectual capital and innovation performance (Agostini et al. 2017), resilience (Daou et al. 2019), organizational agility (Cegarra-Navarro and Martelo-Landroguez 2020), and company growth (Troise et al. 2020). In particular, human capital is recognized as a fundamental resource for start-ups since the founders’ skills and competences constitute the basic element on which a start-up company is founded.

A wide range of entrepreneurs’ characteristics such as technical skills, mindset, and business know-how are success factors for newly-founded companies (Cassar 2007; Vey et al. 2017). Prior studies have suggested that relational capital is an important determinant of the performance of SMEs and start-ups. The entrepreneur’s personal network often plays a crucial role in allowing the company to access resources that are not always easily accessible to SMEs (Gronum et al. 2012). Organizational capital represents the most complex issue for new ventures, mainly due to the short time available to internalize knowledge in companies not yet consolidated (Hormiga et al. 2011). SMEs able to leverage their structural capital have a greater chance of success since this represents a value driver in maintaining a competitive advantage (Martın-de-Castro et al. 2006).

In summary, antifragility is a property that some organizations demonstrate in response to a shock, characterized by: (1) the ability to adapt to a changed context, and (2) the tendency to improve company performance instead of seeking the preservation of the conditions prior to the crisis. Start-ups are organizations that, by nature, have not yet adapted to the environment. Striving to maintain the pre-shock state makes no sense to them. In the face of change generated by a crisis, they are expected to activate intellectual capital, their absorptive capacity, and slack resources as far as they are available to overcome the crisis while, at the same time, achieving a better state of equilibrium.

## Hypotheses development

**Fig. 1:**
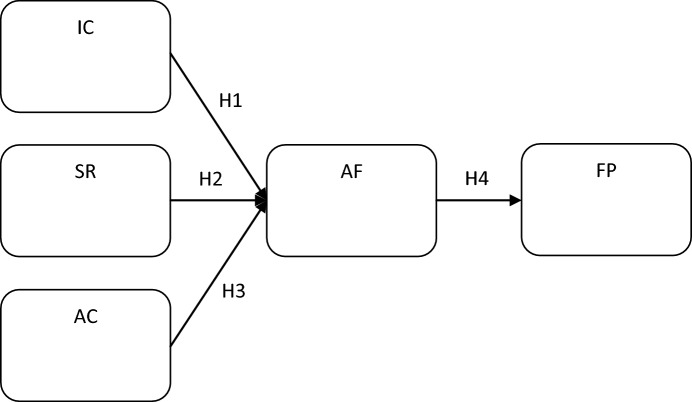
Research model

Figure 1 shows the research model adopted by this research. We hypothesize the presence of three antecedents predicting start-ups’ antifragility. They are intellectual capital, considered in terms of its structural, human, and relational components (Edvinsson et al. 1997); slack resources, understood as the availability of resources, in particular tangible resources in greater quantities than strictly necessary (Leuridan and Demil 2021); and absorptive capacity, or the capacity closely linked to companies’ learning (Cohen and Levinthal 1990). The model also proposes a direct relationship between a start-up’s antifragility and its performance, in particular the financial aspects.

### Intellectual capital and antifragility in start-ups

Firms create and apply their knowledge in an effort to achieve superior performance (Cohen and Levinthal 1990; Grant 1996). Subramaniam and Youndt (2005) evidenced that knowledge is accumulated by the firm through a combination of human (e.g. individuals), relational (e.g. networking), and organizational capital (e.g. the systematization of knowledge through systems and processes). These forms of capital have been acknowledged as intellectual capital (Youndt et al. 2004; Reed et al. 2006). The impact of intellectual capital on a firm’s performance has been widely recognized in the literature. Some characteristics of start-up companies such as R&D attitude, lean organizational structure, creativity, and flexibility make intellectual capital particularly relevant for start-ups. In fact, several works have emphasized the role of intellectual capital in improving SMEs’ innovation performance. For example, McDowell et al. (2018) found a positive relationship between intellectual capital and organizational performance upon analyzing a set of 460 small businesses. Agostini et al. (2017) found that SMEs’ investments in intellectual capital are inextricably linked with innovation performance.

According to Subramaniam and Youndt (2005), a simple organizational structure favors employees’ abilities to network, collaborate, and share knowledge. Start-up firms founded by individuals who have a higher level of prior work experience (Parker 2013) and relevant personal networks (Gronum et al. 2012) exhibit superior innovation performance.

Typical start-ups’ characteristics such as flexibility, technical knowledge, and creativity are particularly crucial when facing crises and external negative events (Frare and Beuren 2021). Intellectual capital is a relevant capacity to possess and apply to overcome difficulties, serving as a means to ensure a company’s survival and achieve innovation (Verbano and Crema 2016; Franco et al. 2021). Companies with strong human capital can be better prepared when encountering sudden disruptive events (Caputo et al. 2019). Employees with high expertise and experience are able to assist the firm in quickly recovering from any catastrophic events and adapting to the new business environment (Martın-de-Castro et al. 2011).

A high level of human capital is tightly linked with a high personal commitment while the possession of soft skills and psychological values (Ahmed et al. 2019) allows companies to become more resilient when facing disruptions (Alfalla-Luque et al. 2013). Moreover, embracing sharing knowledge and the possession of coordination skills enable companies to reduce the effects of a crisis and overcome the long-lasting impact of the negative event (Mubarik et al. 2021). Since intellectual capital favors the strategic resilience, innovation, and competitive advantage of SMEs and start-ups, we formulate the following hypothesis:

#### H1

Intellectual capital has a positive impact on antifragility in start-ups.

### Slack resources and antifragility in start-ups

Research on resilience in organizations often emphasizes the role of reserve resources in ensuring the ability to survive crises (Grandori 2020; Leuridan and Demil 2021). Works dealing with the impact of organizational slack resources on resilience have mainly focused on large firms, finding that slack resources act as a buffer in periods of crisis (Tognazzo et al. 2016).

Research on start-ups has underlined that slack resources are important in favoring the adaptability of these organizations (de Jong et al. 2021). This positive impact of slack resources on adaptability seems to be stronger in the turbulent contexts associated with large crises (Bicen and Johnson 2014). According to De Carolis et al. (2009), slack resources are crucial to absorb, bounce back, and grow in adverse times. Manfield and Newey (2017) argued that, in entrepreneurial companies, the reaction to a crisis differs depending upon the presence/absence of slack resources. As a consequence, the following hypothesis is formulated:

#### H2

The availability of slack resources has a positive impact on antifragility in start-ups.

### Absorptive capacity and antifragility in start-ups

Absorptive capacity is an important factor in organizational learning processes and has been found to be relevant in several cases of adaptation to change (e.g. Liu et al. 2013). In the case of crises, absorptive capacity has been found to impact positively on resilience, in particular on operational resilience at the supply chain level (Gölgeci and Kuivalainen 2020). Absorptive capacity plays an important role in reducing the vulnerability of start-up companies and improving their ability to exploit shared resources (Yuan et al. 2022). Absorptive capacity has been found to have a positive impact on innovation and adaptation in start-ups (Carvalho et al. 2021). Accordingly, the following hypothesis is posited:

#### H3

Absorptive capacity has a positive impact on antifragility in start-ups.

### Antifragility and financial performance in start-ups

Conceptually, the main difference between resilience, in particular evolutionary resilience (Simmie and Martin 2010) and antifragility, is that improvement in an organization is inherent in the concept of antifragility (Ramezani and Camarinha-Matos 2020). In this paper, an antifragility measure is formulated to express this idea of strategic improvement. The improvement of a firm’s strategic position is expected to have a significant impact on financial performance. This is obviously true for start-ups (e.g. Park et al. 2020). The theoretical model developed and tested also includes the following hypothesis:

#### H4

Antifragility has a positive impact on financial performance in start-ups.

## Methodology

### Research setting and data collection

The object of this study is to assess Italian start-ups, an important strategic sector of the economy of the country (Pini and Rinaldi 2021). We investigated the reaction of start-ups to the crisis generated by the COVID-19 pandemic, which affected the world and Italy, in particular from the early months of 2020. In the study of the effects of the crisis, the Italian case is particularly interesting. Italy was the first western country to face the emergency from COVID-19 and to adopt the lockdown measure. The effects on businesses. Many measures undertaken for the first time in Italy have served as models for other countries. To gain a better understanding of the relationship between antifragility and intangible resources, tangible surplus resources (or slack resources) and the absorptive capacity of innovative start-up companies, we purposively sampled cases of start-ups, according to the following criteria (Junge et al. 2022):

1. They were in business at least one year before the start of the COVID-19 pandemic crisis
2. They are based in Italy and
3. Complete and clear information regarding these companies is available.

The first criterion allowed us to observe these differences pre- and post-crisis. The reason for focusing on cases from a single country, i.e. Italy, is that different national business systems (e.g. government support and specific funding to combat the pandemic crisis) could affect the reaction to the crisis differently. Finally, the availability of complete and clear information on start-ups allowed us to get in touch with these companies and administer the questionnaire correctly.

We carried out our research using a sample of 181 start-ups in Italy, extracted from a specific register (accessible at https://start-up.registroimprese.it/). Not all the companies on the register, however, provided full details. For example, not all the companies’ contacts were available. A final list of 1312 start-ups was obtained from the register at the end of July 2021. Data collection was performed through a self-administered questionnaire sent to these firms (Evans and Mathur 2018).

We first carried out a pilot-study on eight start-ups. This activity was useful to make the proposed questions clearer (Ruel et al. 2016). This activity also allowed us to assess the correctness and completeness of the survey (Podsakoff et al. 2012) and, consequently, refine the questions. At the beginning of the survey, we provided an introductory section to explain the research objective and assure participants’ anonymity. With the aim of involving knowledgeable informers, we addressed the survey to the company CEO (or other comparable figures, e.g. the general manager). Moreover, items related to the different constructs were intermixed throughout the questionnaire. This was useful in reducing common method bias (Podsakoff et al. 2003; Kline et al. 2000).

The survey was launched in July 2021 and closed in September 2021. Of the initial 340 respondents (about 26% of the population), 159 were excluded because their questionnaires were incomplete. The total final number of correct forms was 181, representing an overall response rate of about 14%.

The quality of the data was verified by checking for biases derived from insufficient effort responses (Costa and McCrae 2008). In particular, the presence of “long strings” was controlled, i.e. specific sequences of answers provided by an individual. In our sample, we found that no answer had a string longer than five. This can be interpreted as absence of systematic insufficient effort responding (Huang et al. 2012). Table 2 details the characterization of the sample in terms of industry sector, geographical location, and number of employees.

**Table 2:** Sample characteristics

Industry sector	Service activities and utilities	36%
	ICT	21%
	Manufacturing	18%
	Healthcare	7%
	R&D	4%
	Financial, insurance and banking activities	3%
	Trade and retail	3%
	Agri-food	3%
	Transportation	2%
	Construction	1%
	Real Estate	1%
	Others	1%
Geographical location	North	48%
	Center	27%
	South and Islands	25%
Number of employees	0–4	23%
	5–9	32%
	10–19	27%
	20–49	18%

### Measures

Table 3 presents the measures adopted in this study for which we used a seven-point Likert scale (1 = strongly disagree; 7 = strongly agree). To measure antifragility, we adapted a measure of proactive resilience developed by Jia et al. (2020). Notably, proactive resilience shows several similarities with antifragility. In particular, both properties imply proactiveness in adapting to the new context generated by the crisis. However, proactive resilience is neutral with regard to the nature of the adaptation. In other words, for an organization to be considered proactively resilient, it is not necessary that its performance improve after the shock: it is sufficient that the system is able to survive. Antifragility, in contrast, assumes an improvement of the subject’s situation after a disaster.

**Table 3:** Measures

Variable		Measure		Source
SR	Slack resources	SR1	Our company usually has adequate resources available in the short run to fund its initiatives	Essuman et al. (2020)
		SR2	We are often able to obtain resources at short notice to support new strategic initiatives	
		SR3	We often have substantial resources at the discretion of management for funding strategic initiatives	
		SR4	Our company usually has a reasonable amount of resources in reserve	
FP	Financial performance	FP1	Customer retention	Bhatti et al. (2021)
		FP2	Sales growth	
		FP3	Profitability	
		FP4	Return on investment	
		FP5	Overall financial performance	
AC	Absorptive capacity	AC1	Your company has high capability of searching for external information and knowledge	Hsu and Fang (2009)
		AC2	Your company has high capability of identifying the usefulness of external information and knowledge	
		AC3	Your company has high capability of utilize newly acquired knowledge	
HC	Human Capital (HC)	HC1	employee empowerment is high in my company compared to competitors	Hsu and Fang (2009)
		HC2	employees in my company have excellent professional skills compared to competitors	
		HC3	the company provides well-designed training programs compared to competitors	
		HC4	the employees of my company have unique and new ideas compared to competitors	
SC	Structural Capital (SC)	SC1	IT infrastructure components are standardized	Hsu and Fang (2009)
		SC2	Connectivity of IT tools within the firm are adequate	
		SC3	Connectivity of IT tools across the supply chain are adequate	
		SC4	Data is easily sharable within and across the firm	
		SC5	Application systems are highly modular	
RC	Relational Capital	RC1	We work with our partners to solve problems	Ojha et al. (2014)
		RC2	Our partners are flexible in response to requests we make	
		RC3	Our partners make an effort to help us during emergencies	
		RC4	When an agreement is made, we can always rely on our partners to fulfill all the requirements	
AF	Antifragility	AF1	We were able to quickly recognize one or more new business opportunities or possibilities to improve our competitive position	Adapted from Jia et al. (2020)
		AF2	We were able to gather and interpret information on new business opportunities or possibilities to improve our competitive position	
		AF3	We were able to quickly identify, formulate, and evaluate a set of possible responses to disruption	
		AF4	We were able to quickly organize a response team of key personnel	

As a consequence, the measure was modified by reformulating the items to indicate an improvement in the situation of the start-up after the COVID-19 crisis. A short introductory paragraph explained that the answers should refer to the reaction of the company, starting from March 2020. A perception-based measure adopted from Bhatti et al. (2021) was used to measure financial performance. Respondents were asked to evaluate their financial performance compared to competitors after the lockdown in 2020 via five items (see Table 3).

To measure the other constructs in our model, we employed measures proven reliable in previous studies; these are reported in Table 3. In particular, to measure intellectual capital (IC), we used a second-order latent construct resulting from three different components, human capital (HC) (Hsu and Fang 2009), structural capital (SC) (Hsu and Fang 2009), and relational capital (RC) (Ojha et al. 2014).

The values of the tests for validity and reliability are reported in Table 4.

**Table 4:** Reliability and validity of the measures

		OL	Cr. Alpha	rho_A	CR	AVE	R^2^
SR	SR1	0.773	0.723	0.722	0.826	0.544	–
	SR2	0.700					
	SR3	0.810					
	SR4	0.659					
AC	AC1	0.851	0.707	0.722	0.838	0.636	–
	AC2	0.848					
	AC3	0.681					
HC	HC1	0.922	0.827	0.833	0.898	0.746	–
	HC2	0.869					
	HC3	0.795					
SC	SC1	0.652	0.903	0.920	0.930	0.729	–
	SC2	0.925					
	SC3	0.874					
	SC4	0.893					
	SC5	0.897					
RC	RC1	0.705	0.814	0.815	0.878	0.645	–
	RC2	0.842					
	RC3	0.850					
	RC4	0.806					
AF	AF1	0.895	0.915	0.916	0.940	0.798	0.376
	AF2	0.926					
	AF3	0.903					
	AF4	0.848					
FP	FP1	0.751	0.937	0.944	0.953	0.802	0.497
	FP2	0.927					
	FP3	0.944					
	FP4	0.920					
	FP5	0.922					

*OL* outer loadings, *CR* composite reliability, *AVE* average variance extracted

### Data analysis and results

The analysis for this study was carried out using the partial-least squares approach to structural equation modeling (PLS-SEM). This was performed using SmartPLS 3.3.3 software (Ringle et al. 2015). We decided to adopt the PLS-SEM technique as it is suitable for small samples (Willaby et al. 2015) and exploratory studies (Hair et al. 2019). This approach is also suggested for datasets with a small number of indicators for each latent variable (Hair et al. 2019).

Following Koch and Lynn (2012), we performed a full-collinearity analysis to assess the presence of a common method bias. We calculated the variance inflation factor (VIF) values between the latent variables; the value of the higher inner VIF is 2.04 for absorptive capacity and firm performance. Since we found a value of the higher inner VIF lower than 3.3, we can conclude that our model presents a low risk of common method bias. PLS-SEM was organized in two main steps: (1) evaluation of the quality of the measurement model, and (2) evaluation of the structural model’s predictive power (Henseler et al. 2009; Hair et al. 2016).

### Measurement model

We tested for the reliability indicator in order to check the quality of the outer model. We found that no item presents an outer loading lower than the minimum value of 0.6 (Chin 1998; Henseler et al. 2009). We also checked the composite reliability (Hair et al. 2016), Dillon-Goldstein’s rho (Chin 1998), and Cronbach’s alpha (Hair et al. 2016). All values are higher than 0.7; hence, the constructs can be considered reliable. Moreover, we verified the average variance extracted (AVE) and found that the values are always higher than 0.50 (the lowest one is 0.544 for slack resources). Finally, we checked the constructs’ discriminant validity by means of the cross-loading approach proposed by Ravand and Baghaei (2016) (Table 5). Overall, the measurement model validity tests all yielded good results, as detailed in Tables 4 and 5.

**Table 5:** Discriminant validity

ITEM	AC	AF	FP	HC	IT	RC	SLK
AC1	**0.851**	0.475	0.463	0.407	0.401	0.433	0.562
AC2	**0.848**	0.434	0.356	0.393	0.385	0.370	0.434
AC3	**0.681**	0.385	0.319	0.599	0.469	0.404	0.387
AF1	0.449	**0.895**	0.616	0.364	0.341	0.330	0.417
AF2	0.452	**0.926**	0.645	0.343	0.397	0.313	0.470
AF3	0.484	**0.903**	0.606	0.409	0.455	0.305	0.444
AF4	0.549	**0.848**	0.653	0.436	0.471	0.408	0.460
FP1	0.422	0.529	**0.751**	0.303	0.462	0.305	0.464
FP2	0.413	0.667	**0.927**	0.324	0.424	0.350	0.519
FP3	0.423	0.680	**0.944**	0.310	0.403	0.354	0.559
FP4	0.475	0.645	**0.920**	0.315	0.397	0.419	0.565
FP5	0.426	0.633	**0.922**	0.302	0.404	0.405	0.624
HC1	0.582	0.403	0.312	**0.922**	0.460	0.430	0.340
HC2	0.466	0.327	0.256	**0.869**	0.347	0.445	0.266
HC3	0.434	0.397	0.328	**0.795**	0.415	0.436	0.291
IT1	0.349	0.182	0.235	0.294	**0.652**	0.216	0.292
IT2	0.501	0.415	0.388	0.466	**0.925**	0.364	0.405
IT3	0.410	0.419	0.423	0.414	**0.874**	0.384	0.347
IT4	0.423	0.426	0.403	0.390	**0.893**	0.340	0.353
IT5	0.521	0.504	0.495	0.440	**0.897**	0.415	0.433
RC1	0.512	0.365	0.347	0.398	0.378	**0.705**	0.361
RC2	0.374	0.292	0.304	0.467	0.346	**0.842**	0.366
RC3	0.362	0.281	0.290	0.342	0.302	**0.850**	0.375
RC4	0.370	0.286	0.375	0.409	0.283	**0.806**	0.472
SLK2	0.370	0.285	0.469	0.203	0.285	0.312	**0.773**
SLK3	0.377	0.304	0.393	0.094	0.242	0.290	**0.700**
SLK4	0.292	0.400	0.523	0.184	0.291	0.299	**0.810**
SLK5	0.626	0.438	0.403	0.465	0.410	0.492	**0.659**

The data in bold are the items’ outer loadings to their constructs

### Structural model and hypotheses testing

We analyzed the structural path coefficients retrieved through the bootstrapping method based on 5,000 resamples (Hair et al. 2016). Then through the *R*^2^ value, we evaluated the related predictive power of the constructs. Table 6 shows the *R*^2^ values of the endogenous constructs. We found a moderate predicting power for financial performance (0.309) and a low predicting power for antifragility.

**Table 6:** Hypothesis testing

HP	Original sample (O)	Sample mean (M)	Standard deviation (STDEV)	T Statistics (|O/STDEV|)	*P* values
AC—> AF	0.237	0.238	0.101	2.363	0.018
AF—> FP	0.707	0.711	0.048	14.801	0.000
IC—> AF	0.269	0.265	0.114	2.357	0.018
SLK—> AF	0.221	0.236	0.069	3.201	0.001

**Fig. 2:**
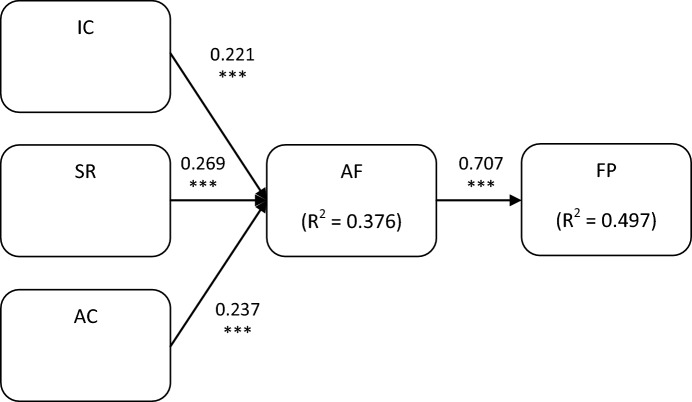
Results of the model evaluation

All the hypotheses proposed in this paper received support based on the results of our research. In particular, IC (0.221***), SR (0.269**), and AC (0.237***) have an impact on antifragility. At the same time, antifragility has a significant impact on financial performance (0.707***). The results of the tests of the hypotheses are reported in Table 6, and the corresponding data can be found in Fig. 2.

## Discussion

This research attempted to advance the current body of knowledge on the antecedents and outcomes of antifragility. In spite of the growing interest in both resilience and antifragility (Ramezani and Camarinha-Matos 2020), our understanding of the antecedents of these two variables, in particular the second one, is still limited (Nikookar et al. 2021). In addition, the number of studies focusing on start-ups is extremely limited.

Specifically, we investigated the impact of three antecedents of antifragility in the context of start-ups: intellectual capital, slack resources, and absorptive capacity.

The analysis of the literature on resilience, in particular adaptive resilience, suggests that these three variables have a positive impact on the capability of firms to adapt in times of disruptive change. These findings are in line with previous research that demonstrates the positive impact of slack resources in turbulent contexts (Bicen and Johnson 2014) and the importance of absorptive capacity in improving company performance (Liu et al. 2013).

We found that intellectual capital and absorptive capacity play an important role in the development and success of start-ups. This confirms the previous findings reported by Carvalho et al. (2021). The analysis of the data provided validation for our theoretical arguments and fully supported the hypotheses. These three variables do positively influence antifragility in start-ups, in line with our *H1*, *H2*, and *H3*. These results confirm insights from prior studies, extending to start-ups the validity of relationships already studied for other types of companies.

Our findings evidence the importance of antifragility in determining firm performance as perceived by entrepreneurs. The empirical evidence supports *H4*, thus indicating that antifragility enhances the performance of start-ups. With reference to *H1*, our findings confirm that human, structural, and social capital can be seen as part of an overall construct (Subramaniam and Youndt 2005). Moreover, we found that this overall dimension, called intellectual capital, has a positive impact on antifragility and, through antifragility, on performance.

The existence of a latent construct, called intellectual capital, has a positive impact on performance while its individual components do not exert a statistically significant influence. This suggests that the three components are somehow interchangeable or complementary (Shakina and Barajas 2014). It is not necessary for all of them to have high values for start-ups to be antifragile. What matters is the overall level of intellectual capital.

This work also confirms findings from past studies that suggests the importance of slack resources for developing resilience in the face of disruptions (Leuridan and Demil 2021) for the adaptation processes of start-ups in general (de Jong et al. 2021) and specifically in the context of turbulent environments (Bicen and Johnson 2014). Our work thus contributes to broadening the literature stream by extending their findings to the case of antifragility in start-ups. Although start-ups are characterized by the scarcity of resources (Gimenez-Fernandez et al. 2020), careful management can lead to a limited redundancy that favors antifragility.

The literature on absorptive capacity underlines its importance for the ability to adapt to change (Liu et al. 2013). In particular, it contributes to overcoming crises by supporting resilience (Gölgeci and Kuivalainen 2020). In line with this argument and with reference to new, technology-based firms, our findings suggest that absorptive capacity also supports antifragility, thus contributing to successfully overcoming crises by not only adapting to new contexts, but also improving the competitive position of start-ups.

The relevance of antifragility seems to be confirmed by the fact that, in the perception of the interviewed entrepreneurs, antifragility has a positive impact on financial performance. This seems to confirm the notion that antifragility, i.e. the capability driven by the investigated antecedents, not only contributes to adapting to new contexts determined by a crisis, as is the case for resilience (Simmie and Martin 2010), but also to the improvement of a start-up’s performance after a shock (Ramezani and Camirinha-Matos 2020).

Consistent with Munoz et al. (2022), our research confirms the idea that antifragility can be seen as a property or capability of some organizations, resulting from a combination of resources and capacities. These resources are both tangible and intangible. The availability of resources, together with the ability to learn and adapt, supported by absorptive capacity, allow start-ups to improve their strategic position after a shock. Our findings contribute to the literature by showing that antifragility allows companies and start-ups in particular, to survive the challenges of large disasters (Rialti et al. 2019) while also enabling them to obtain superior performance.

## Conclusion, limitations, and avenues for future research

This study aimed to address the research question “What factors support antifragility in innovative start-ups?” The results suggest that antifragility can be built by investing in a combination of tangible and intangible resources, as well as learning capabilities. The antecedents found to positively impact antifragility are: intellectual capital, slack resources, and absorptive capacity. In turn, antifragility is positively associated with improved financial performance, thereby highlighting its relevance for start-ups to successfully overcome crises.

This work further contributes to the current academic debate on the role of antifragility and its antecedents, specifically extending the debate to the case of start-ups. We have presented and empirically tested a theoretical model consisting of three drivers of antifragility and one outcome. The findings add to the body of research on antifragility (Ramezani and Camirinha-Matos 2020) by focusing on a type of company little explored in the literature, namely start-ups.

This paper has several practical implications to offer. Overall, the research provides useful guidelines for start-ups and their management in the context of disruptive shocks. Evidence suggests that intellectual capital acts as an enabler of antifragility in start-ups. However, a certain amount of slack tangible and financial resources is necessary to exploit crises and improve the strategic position of the company. Founders and managers of start-ups should be aware of the need to build a balanced mix of resources for their start-ups to become antifragile. Learning capabilities, driven by absorptive capacity (Cohen and Levinthal 1990), seem to be a necessary precondition for antifragility. While learning is a normal attitude for start-ups (Steiber et al. 2020), it is critical to understand what learning domains are most important. This could be the objective of future studies.

Our study also suggests that antifragility is associated with an improvement in performance, thus underlining its importance. This finding is relevant to governments and policymakers as start-ups contribute to build the business ecosystems of the future (Pini and Rinaldi 2021). These actors should attempt to create helpful conditions favoring the growth and development of start-ups, paying particular attention to factors affecting their performance. As crises can jeopardize the growth of start-ups, nations and economies able to build antifragility in start-ups could be in a position to exploit crises and improve their overall competitiveness. Thus, the identification of conditions enabling companies to be antifragile, and in turn improve their performance, enables the formulation of more effective policies that would stimulate and encourage specific programs and practices.

This study has some limitations. First, this research is focused on a single country, namely Italy. Although Italy is an interesting case, as it was the first Western country to face the COVID-19 emergency, resulting in a case study for all the others, it is also true that the responses of the states have been different. Factors such as national culture, the country's economic structure, political choices, may have influenced the response of start-ups. Future studies must analyze the impact of these contingent variables on the antifragility of start-ups. Further research could investigate other contexts to confirm our findings and extend the current results. Second, other measures and scales could be used to fully capture a broader scope. For example, the scale adopted for antifragility is an adaptation of a measure for resilience (Jia et al. 2020). Specific measures for antifragility thus need to be developed (Größler 2020). A useful set of measures has been collected, in the case of resilience, by Hilmann and Guenther (2021). These measures can serve as a model for measures of antifragility. Third, the composition of the sample in terms of industrial sectors, reflects the overall percentages present in the Italian register of start-ups. Some sectors are under-represented. Future studies should analyze in more detail the specific responses of start-ups to disaster events in different sectors.

These two authors conducted a review of existing measures, highlighting how most measures are based on ex-post evaluations. However, though their analysis, they identified factors and measures that can help predict resilient behaviors. A similar approach is desirable in the case of antifragility. Further, the measure for financial performance adopted in the present study is perception-based. Objective measures (e.g. return on investments, profits, market value) should be used in future studies. Furthermore, our study did not consider the long-term effects of large-scale crises. Future studies could therefore consider the long-term effects of the COVID-19 pandemic for start-ups. In particular, longitudinal in-depth case studies could follow a few start-ups from near birth to death or to a fully-fledged company. Alternatively, the long-term effects of past crises could be studied. Further studies may also consider new types of organizations or other countries. Of note, studies based on the corporate history research method could be conducted to analyze the effects of past crises.

## Data Availability

The datasets generated and analyzed during the current study are available from the corresponding author on reasonable request.
